# 

**Published:** 1889-07

**Authors:** 


### BOOK NOTICES.

EUROPEAN GLIMPSES AND GLANCES, by J. M. Emerson. This is the author's second book of transatlantic notes and observations, and a very readable and instructive one it is, too. It requires genius to make a book of travels over fields which have been so often visited and described, interesting to the general reader, but Mr. Emerson has done this in a way to be commended. He has not merely skimmed the surface, but by making himself familiar with the domestic ways and home life of the people, he has been able to acquaint us with many of those everyday happenings which the casual observer passes by unnoticed. An excellent feature of the book is its many and accurate wood cuts. There is also a clever electro-engraving of the author, which makes it valuable as a souvenir of one of our most esteemed citizens. New York. Canell & Co.

LIGHT ON THE PATH.—A treatise on the occultism of the far east. Theosophical Book Co., 110 Tremont street, Boston. The object of this compact little book is stated to be "To put practical occultism into words." Its first three precepts are: "1, Kill out ambition; 2, Kill out desire of life; 3, Kill out desire of comfort." We think it hardly worth our while to attempt a review of a work whose precepts are so utterly inconsistent with the duties of citizenship; the family and the state. If, as it is taught, "the soul must have laid aside the emotions of humanity, must have secured a balance which cannot be shaken by misfortune before its eyes can open upon the super-human world," then we would prefer to remain as we are, to being encased in this impenetrable armor of selfishness. The whole effort seems to be to unfit one for this world and its conditions. No example is of any use to man which is not suited to his requirements, as one of a common family. An adept, in a true sense, is little else than an emasculated monstrosity, and the fewer we have of them the better. He may be able to rob himself of all the attributes of manhood, subdue his passions, deny his appetites, crush out benevolence and destroy sympathy, but, after all, of what use is he to his kind, or even to himself. He is a kind of mummified specimen of his race, adapted, perhaps, to a museum of freaks, but utterly worthless for any practical use. The man who put two billiard balls in his mouth, the other day, and his fellow, who swallowed an open-bladed knife on a wager, are of the same price. For ourselves, we would none of it.

OUR thanks are due for the following interesting treatises and papers:

REPORT OF THE JACKSONVILLE Auxiliary Sanitary Association. Epidemic of 1888, 276 pp., with appendix, edited by Charles S. Adams. This pains taking report abounds in facts and statistics of great value to the medical profession.

THE Radical cure of Hernia, by Thomas W. Kay M. D., Scranton, Pa.

OPINIONS ON CREMATION. The New York Cremation Society. Morse Building 140 Nassau st., N. Y. City. James B. Brown, Secretary.

DISCUSSION OF THE MEDICAL BILL. Edited by F. J. McKenzie, Oskosh, Wis.

MEANS OF PURIFYING DRINKING WATER, by Charles G. Currin M. D. N. Y. City,

THE CENTURY MAGAZINE, is still in the lead of American monthlies, and in the excellence of its wood cuts, leads the world. The Lincoln biography, and the Siberian papers are unflagging in interest. These alone are worth more than the year's subscription. The Century Co., N. Y. City.

